# Atypical Immunoglobulin A Vasculitis in a Pediatric Patient With Ulcerative Colitis

**DOI:** 10.14309/crj.0000000000001145

**Published:** 2023-09-05

**Authors:** Tejas S. Desai, Roman Jurencak, Asha Nair, Nicholas Carman

**Affiliations:** 1 Division of Pediatric Medicine, Children's Hospital of Eastern Ontario, Ontario, Canada; 2 Department of Pediatrics, Faculty of Medicine, University of Ottawa, Ottawa, Ontario, Canada; 3 Division of Rheumatology, Children's Hospital of Eastern Ontario, Ontario, Canada; 4 Division of Gastroenterology, Hepatology and Nutrition, Children's Hospital of Eastern Ontario, Ontario, Canada

**Keywords:** pediatric, vasculitis, ulcerative colitis, IgAV, immunoglobulin A vasculitis

## Abstract

Rates of pediatric inflammatory bowel disease and biologic therapy use continue to rise. Consequently, specialists and generalists should recognize potential complications and side effects. We report the unique case of an adolescent with ulcerative colitis (UC) on vedolizumab presenting with severe abdominal pain, hematochezia, and subsequent purpura. After extensive investigation and a complex clinical course, diagnosis of atypical immunoglobulin A vasculitis was made. This is the first pediatric case of vasculitis in a patient with UC on vedolizumab and only the second reported case overall in UC. This case illustrates the emerging diagnostic challenge of distinguishing inflammatory bowel disease treatment complications from other common pediatric conditions.

## INTRODUCTION

Ulcerative colitis (UC) is a form of inflammatory bowel disease (IBD) characterized by relapsing and remitting inflammation involving the colon.^1^ Canada has one of the highest rates of pediatric IBD worldwide, with recent incidence data estimating 9.68 cases per 100,000 population.^2^ Various treatment options exist for pediatric patients, with biologic therapies increasing in use given poor disease control with conventional therapies.^1^ Vedolizumab (VDZ) is a newer, gut-selective monoclonal antibody against integrin α_4_β_7_ approved for moderate-to-severe UC and Crohn's disease (CD) in adults^3^ after efficacy was demonstrated through the GEMINI program.^4^ It has been administered off-label in refractory pediatric UC in Canada through the Canadian Children IBD Network (CIDsCANN) since 2016 (NCT02308917).

Immunoglobulin A vasculitis (IgAV), formerly Henoch-Schönlein purpura, is typically a self-resolving condition characterized by palpable purpura, gastrointestinal symptoms, arthritis, and renal dysfunction. Cutaneous vasculitis is a known side effect of most biologic therapies, especially tumor necrosis factor (TNF) inhibitors, but only a few case reports exist with VDZ. Considering this overlap between biologics, vasculitis, and immune-mediated disorders, diagnosis and management of such presentations can be challenging.

In this report, we outline only the second case of IgAV in UC and the first pediatric case of vasculitis in a patient with UC on VDZ.

## CASE REPORT

A 14-year-old adolescent boy with UC was admitted to hospital in January 2021 after 1 week of severe abdominal pain, increasing vomiting, and melena, atypical of his usual UC symptoms. UC was diagnosed in March 2017. Colonoscopy identified severe (Mayo 3) left-sided colitis, with histopathology consistent with UC. Induction with intravenous steroids was unsuccessful, prompting escalation to infliximab, in combination with methotrexate (MTX). Given an incomplete response, a switch to VDZ was made in spring 2018, resulting in successful and sustained remission. The patient remained in steroid-free clinical (Pediatric Ulcerative Colitis Activity Index 0) and biochemical (fecal calprotectin 109 μg/g with normal range <250 μg/g) remission on standard dosing VDZ (300 mg every 8 weeks), in combination with MTX (12.5 mg per os weekly). His last infusion was 5 days before admission.

**Figure 1.**
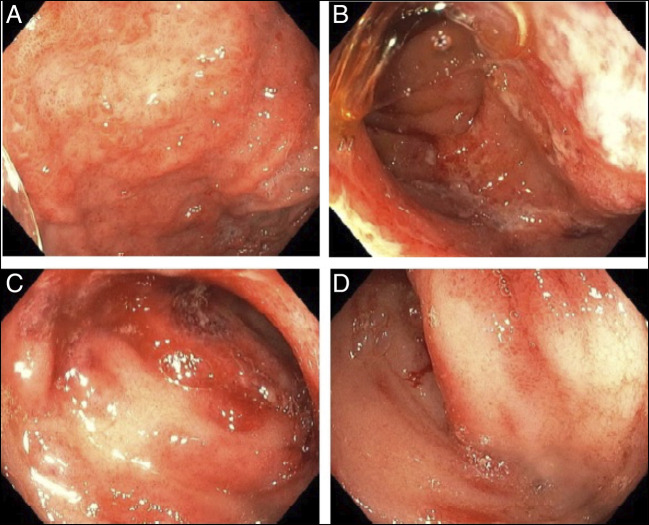
Gastric (A), duodenal (B), and ileal (C, D) endoscopic findings.

Laboratory investigations at admission demonstrated a white blood cell count (WBC) of 18 × 10^9^/L, C-reactive protein (CRP) of 62 mg/L, albumin of 29 g/L, and fecal calprotectin of 2,470 μg/g. Routine biochemistry and urinalysis were unremarkable. Extensive infectious testing of stool, blood, and urine for viruses and bacteria was negative. Magnetic resonance enterography demonstrated a prominent loop of jejunum. Endoscopy revealed severe gastric, duodenal, and ileal inflammation suggestive of a vasculitic process (Figure 1), which correlated with histology, suggesting small-vessel vasculitis.

**Figure 2.**
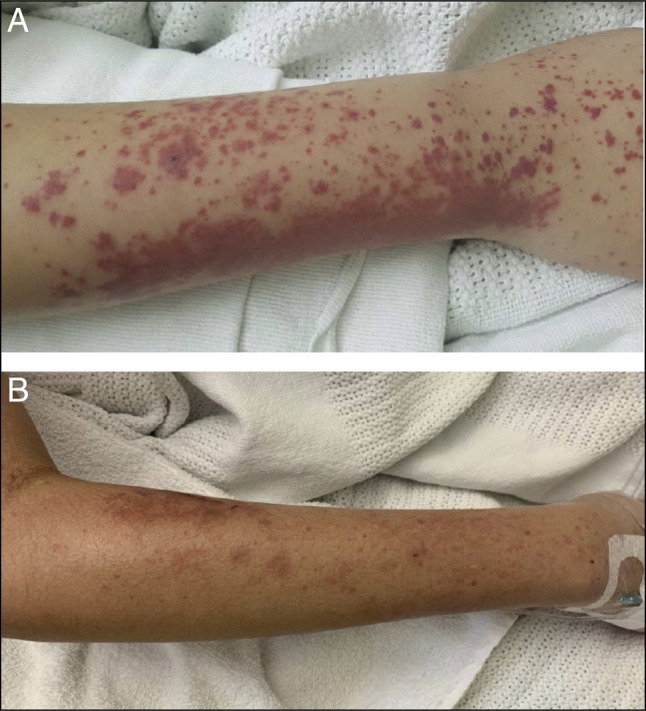
Upper arm purpura at onset (A) and at discharge (B).

On day 4 of admission, the patient developed purpura to both upper arms (Figure 2) with ongoing, intermittent abdominal pain and a corresponding rise in CRP and WBC. Rheumatology, dermatology, and infectious diseases were consulted. Antinuclear antibodies, antineutrophilic cytoplasmic antibodies, immunoglobulins, and complement and coagulation studies were all subsequently normal. Skin biopsy was not completed because of significant pathergy and concerns for subsequent pyoderma gangrenosum. Consensus was of severe inflammation from an unusual presentation of leukocytoclastic vasculitis because of IgAV. Treatment with topical betamethasone valerate and intravenous methylprednisolone (1 mg/kg) was commenced.

After initial improvement, abdominal pain and hematochezia worsened by day 9 of admission, coinciding with increases in inflammatory markers (CRP 40 mg/L, WBC 27 × 10^9^//L, platelets 544 × 10^9^/9/L). Urgent ultrasound and computed tomography of the abdomen demonstrated interval progression of bowel wall thickening involving the entire ileum and distal jejunum. Treatment with intravenous immunoglobulin and high-dose methylprednisolone (1 g/d) was started. Patient-controlled analgesia and peripheral total parenteral nutrition were started.

On initiation of pulse steroids, rapid clinical, biochemical, and imaging improvement was observed. Oral steroids were initiated once full diet was resumed. At discharge after 20 days, all laboratory investigations were improving or normalized. Microscopic hematuria with normal renal function and blood pressure was noted 1 month after discharge and continued to be monitored by nephrology. At 1 year, the patient remained well with resolved hematuria and achieved complete clinical and biochemical remission of his UC (Pediatric Ulcerative Colitis Activity Index 0, calprotectin 25 μg/g), while remaining on VDZ and MTX at the same dose.

## DISCUSSION

With increasing use of biologic agents and predisposition for other immune-mediated conditions in IBD, vasculitis is an important clinical consideration for subspecialists and pediatricians alike.

IgAV is a small-vessel vasculitis, typically affecting children younger than 10 years (∼90% cases).^5^ Diagnostic criteria were outlined in 1990 and updated in 2010; however, the exact pathophysiology remains unclear, other than the significant role of IgA.^5^ A genetic predisposition, especially among East Asians, and environmental factors also seem to affect disease onset. How underlying autoimmune disease or biologic use alters the pathophysiology of IgAV, if at all, remains unknown. This, however, highlights the need for heightened vigilance on patients with multiple risk factors.

Vasculitic complication rates vary among available biologic agents, with cutaneous findings ranging between 20% and 85% in published reports.^6,7^ Anti-TNF agents are most commonly implicated and may occur years after treatment.^6,7^ Vasculitis reports are more common in patients with Crohn's disease than UC and rare among those on VDZ, which may reflect its gut-selective mechanism, although usage compared with anti-TNF agents is more limited. Pathologic findings typically suggest leukocytoclastic vasculitis.^8,9^ Unfortunately, clinical concerns prohibited skin biopsy in our patient, relying instead on clinician expertise for diagnosis. Unique in our patient was the severity and progression of enteritis despite recent VDZ and steroid treatment, further complicating diagnosis and treatment. Whether this related to an unidentified trigger remains unclear. With rapidly growing biologic options of various mechanisms, practitioners should familiarize themselves with these agents because nonspecialists may increasingly be the first line of care. This is key for timely diagnosis and management of potential complications as well as recognition of drug-independent mechanisms that may manifest in an atypical manner.

In conclusion, we describe the first pediatric case of vasculitis associated with UC on VDZ. The presentation and successful treatment with therapy aimed at IgAV, with subsequent resolution of vasculitis despite ongoing use of VDZ, suggests an independent autoinflammatory phenomenon, rather than a drug effect. Considering the significant morbidity, rising rates of pediatric IBD, and growing use of biologics, this case highlights the important clinical overlap between vasculitis, immune-mediated disease, and biologics that should be considered in related pediatric presentations.

## DISCLOSURES

Author contributions: TS Desai: literature review, drafting and final editing of the manuscript, approval of the final manuscript, and is the article guarantor. R. Jurencak and A. Nair: review and editing of manuscript, and approval of the final manuscript. N. Carman: obtaining patient consent, drafting and review of the manuscript, and approval of the final manuscript.

Financial disclosure: None to report.

Previous presentation: Presented as a poster presentation at the Canadian Association of Gastroenterology Canadian Digestive Diseases Week (CDDW) Conference in March 2022. Abstract was published in the *Journal of the Canadian Association of Gastroenterology* in March 2022.

Informed consent was obtained for this case report.
